# Randomized controlled trial on drowning prevention for parents with children aged below five years in Bangladesh: a study protocol

**DOI:** 10.1186/s12889-015-1823-1

**Published:** 2015-05-11

**Authors:** Mosharaf Hossain, Kulanthayan K. C. Mani, Sherina Mohd Sidik, Hayati KS, AKM Fazlur Rahman

**Affiliations:** Department of Community Health, Faculty of Medicine and Health Science, University Putra Malaysia, 43400 UPM Serdang, Selangor, Malaysia; Department of Psychiatry, Faculty of Medicine and Health Science, University Putra Malaysia, 43400 UPM Serdang, Selangor, Malaysia; Department of Epidemiology, Bangladesh University of Health Sciences, Dhaka, Bangladesh

**Keywords:** Mobile coach, Childhood drowning, Parents, Children below five years of age

## Abstract

**Background:**

Drowning is the third leading cause of death for children aged 0–4 years in many Asian countries, and is a serious but neglected health problem in low and middle-income countries like Bangladesh. The aim of the study is to outline the study protocol of a trial to test the efficacy of a mobile coach based intervention for the prevention of childhood drowning.

**Method/design:**

A two-arm cluster randomized community trial will be conducted to test the efficacy of the mobile coach intervention for childhood drowning on parents with children below five years of age and compared to an assessment only control group. A total of 1680 parents in the villages with children aged below five years of age will participate. The village will be used as a randomized unit, randomly assigned to an intervention group (*N* = 840) receiving the mobile coach based intervention or an assessment only control group (*N* = 840). An individualized mobile coach intervention based on the demographic data and the individual will be developed, and SMSs, audio messages, videos and images about childhood drowning will be sent to the participants of the intervention group over a period of six months. The participants will receive per week one text message (SMS) and image and one video and audio text per month. The primary outcome measure will be increased knowledge and safety awareness, and behaviour practice about childhood drowning assessed at the six-month follow-up, and the secondary outcome measure will be the reduced incidence of childhood drowning in Bangladesh. The study assistants conducting the baseline and the follow-up assessments will be blinded regarding the group assignment.

**Discussion:**

This is the first study testing a fully mobile coach intervention for childhood drowning prevention in Bangladesh. It is hoped that the programme will offer an effective and inexpensive way to prevent childhood drowning among children aged below five years and also increase the awareness of parents concerning the risks to their children from drowning.

**Trial registration:**

ISRCTN13774693, 08/03/2015.

## Background

Drowning is the process of experiencing respiratory impairment from submersion or immersion in liquid; outcomes are classified as death, morbidity and no morbidity [1]. Drowning is the third leading cause of unintentional injury worldwide, and constitutes 7 % of all injury related deaths globally. There are an estimated 388,800 annual drowning deaths worldwide. Among children, drowning accounts for a higher mortality rate than any other, and, unfortunately, the drowning rate in Asia is 20 times higher than in developed countries. The parent’s socio-economic status and lack of education, infants left unsupervised, access to water, and children under 5 years of age, especially males, are the factors that represent the greatest risk from drowning [2]. Drowning is the single leading cause of death after infancy, 50 % of drowning occurs between the ages of 0 to 4 years, 60 % happens between 9 a.m. and 1 p.m., 80 % occurs in ponds, ditches, buckets and drums, 80 % happens within 20 m of the child’s home, and children from large families are twice as vulnerable to drowning than those from small families. In Bangladesh, a child drowns every half hour, with a total of 50 per day and 18,225 during the course of a year [3].

Bangladesh is a largely rural low-income country with most households located near bodies of water. Many children do not know how to swim and families are large, leading to decreased adult supervision. These factors combine to make drowning the leading cause of child death after infancy. The drowning mortality rate is 28.6 deaths per 100,000 child-years in Bangladesh, which is 22 times greater than in the Americas [4–9]. The drowning of rural children aged 1–4 years in Bangladesh was found to be 156.4 per 100,000 (95 % CI 138.5 - 176.6 per 100,000) with boys numbering 175.5 and girls 135.8. The proportional mortality from children drowning in respect of the overall child mortality was about 28 %. Ponds, ditches, buckets and drums were the most frequent places of drowning, and over 40 % occurred in ponds. The risk factors include the sex of the identified child, mother’s age and literacy, family income and ownership of agricultural land by the families [10]. The incidence of drowning among children aged 1 – 4 years old was 157 per 100,000 children-years. The highest rate was 328.1 per 100,000 (95 % CI 254.8 – 421.7) among 1 year old male children. The risk factors include mothers’ age, literacy and family income. Male children from poor families were at great risk of drowning in rural Bangladesh [11–13].

### What are the risks?

Drowning happens in many different ways, and needs a range of prevention strategies to target the biggest risks. The main risk factors are: Lack of physical barriers between people and water, particularly close to home, Lack of supervision of young children, Uncovered or unprotected water supplies and lack of safe water crossings, Lack of water safety awareness and risky behaviour around water, such as swimming alone, Travelling on water, especially on overcrowded or poorly maintained vessels, Flood disasters, whether from extreme rainfall, storm surges, tsunamis or cyclones [14].

### Governmental goals in Bangladesh

Today, injury is the biggest killer of Bangladeshi children between 1–18 years, and the greatest danger is from drowning. The remarkable progress made in the reduction of child mortality from infectious diseases in Bangladesh during the last two decades has meant that injuries are now emerging as a greater threat to child survival. Although the rate of drowning has remained stable since the 1980s, drowning as a proportion of all deaths in children aged 1–4 increased from 9 % in 1983 to 59 % in 2003. Bangladesh Target: To reduce deaths of children under five by two-thirds by 2015; that is, reduce the under-five mortality rate to 48 deaths per 1000 live births [15].

### How can the threat of drowning be reduced?

Drowning can be prevented through targeted prevention strategies, improved community infrastructure (water supply, bridges, levees, etc.), public awareness, appropriate policies and legislation, and research that refines what is seen as best practice and that identifies new drowning prevention measures. Such measures include: Reduced exposure to water hazards through the strategic use of barriers, Comprehensive boating regulations and enforcement, Close and capable adult supervision for young children, Signage and designation of hazardous water bodies, Improved swimming and water safety skills, Timely rescues and resuscitation by a trained bystander or lifesaver through mouth-to-mouth resuscitation and chest compression when needed, Requirements for use of personal flotation devices and Supervision of recreational swimming areas [14].

Therefore, the aim of this study is a trial to test the efficacy of a *MOBILE COACH* based intervention for the prevention of childhood drowning in Bangladesh.

### Research objectives

#### Main objective

- ❖ To develop and implement effective mobile coach intervention on childhood drowning prevention among parents with children’s age below five years in rural areas in Bangladesh

#### Specific objectives

- ❖ To determine the differential between the ages and gender of the children in respect of knowledge, awareness, practice and child drowning.
- ❖ To develop and assess the initial response of a community to an intervention package in terms of acceptability, feasibility and sustainability.
- ❖ To determine the social-demographic factors (educational level of parents, occupation of parents, social-economic status of parents and medical condition), environmental factors (places of residence, time of day, time of season, distance from the house, place of drowning, safety equipment, presence of lifeguard, activity immediately before drowning and mothers activity) and behavioural factors (supervision practices of parents and swimming ability of parents and child) through pre-test and post-test on knowledge, awareness, practice and child drowning.
- ❖ To understand the perception of the community concerning the problem of drowning and its possible implications for designing a preventive programme for the rural areas of Bangladesh.
- ❖ To identify the association between the knowledge, awareness and practice of child drowning with social-demographic variables, environmental variables and behavioural factors.
- ❖ To evaluate the mobile coach for its effectiveness.
- ❖ To determine the risk factors based on social-demographic, environmental and behavioural factors in respect of childhood drowning, knowledge, awareness and practice pertaining to child drowning.

## Method/design

### Design and main hypothesis

**Fig. 1:**
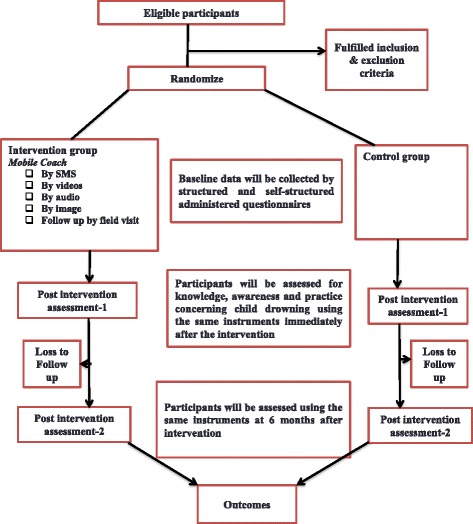
Study design

A two-arm cluster randomised community trial will be conducted to test the efficacy of the mobile coach based intervention for the prevention of childhood drowning among parents with children aged below five years compared to an assessment only control group. In respect of the childhood drowning knowledge, awareness and practice, SMSs, videos, audio messages and images will be sent to participants based on the Ministry of Health and Family Welfare, Bangladesh Government and Centre for Injury Prevention and Research, Bangladesh. The mobile coach will be sent to the participants over a period of six months and data will be gathered at baseline, with a weekly SMS and images, and monthly videos and audio assessment. The main hypothesis is that the integrated intervention will be a more effective and inexpensive way to prevent childhood drowning among children aged below five years, and also increases the parent’s awareness of their children in respect of drowning. At the six-month follow-up, the primary outcome will be the per monthly increase in knowledge, safety awareness and behavioural practice in respect of childhood drowning prevention of the intervention group compared to those in the assessment only control group. The secondary outcome measures will be reducing the incidence of childhood drowning to compare the baseline and end line assessment with the self-efficacy for childhood drowning prevention, and also to determine the risk factors for childhood drowning. An overview of the study design is presented in Fig. 1.

### Participants, setting and procedure

The intervention will be tested on parents with children below five years of age due to the high proportion of drowning of children aged under five years (43 % of deaths among children aged below five years in Bangladesh) [14]. Parents with children aged below five years will be collected from selected rural areas in the Rajshahi district, Bangladesh. College teachers from approximately 15 village schools will be invited to participate with other individuals from their village in a study to test the efficacy of the mobile coach based childhood drowning programme. The participating teachers will schedule one village per hour for screening the eligibility criteria, study information and baseline assessment. The study participants will be recruited by study assistants (graduate students).

The study assistants will invite all the participants from a village to participate in the survey. Furthermore, they will inform other individuals who will be invited to participate in a study to test the efficacy of mobile coach intervention for the prevention of drowning. To decrease the reporting bias, the study assistants will not provide additional information about the purpose of the study before the screening of the eligibility criteria is completed. Afterwards, the participants will be invited to complete the screening. The screening includes the assessment of demographic, environmental, behavioural data, knowledge and awareness about childhood drowning, and ownership of a mobile phone. The inclusion criteria for the study participation are (1) parents who have less than five children, children living with parents, children aged 0–4 and must have a mobile phone in the house. The exclusion criteria are (1) parents who are not available at the time of data collection (after checking the house three times) and children with a physical disability (inability to walk). Subsequently, eligible persons will be informed about the aim of the study, the intervention aims, assessments and data protection. The study information will be provided in paper form by the study assistants. After receiving informed consent, all participants will be given a username and their mobile phone number will be collected.

### Ethical issues

The study protocol has been approved by the University Putra Malaysia medical research ethics committee (No. Reference: UPM/TNCPI/RMC/JKEUPM/1.4.18.1/F1 Date: 11/02/2015). Written informed consent will be obtained from all respondents before collecting data.

### Randomisation and allocation concealment

To avoid spillover effects within the villages, we will conduct a cluster randomised controlled trial using the village as the randomisation unit. Due to the heterogeneity of participants in various disciplines (e.g. gender or educational level), we will use separate randomisation lists for each village (stratified randomisation). Furthermore, for approximate equality of sample size in the study groups, we will use computer generated block randomisation. The study assistants supervising the baseline assessment in the villages will be blinded concerning the group allocation of the village. In addition, group allocation will not be released to the study participants until they provide informed consent, their username, mobile phone number and baseline data. Furthermore, the study assistants conducting the face-to-face interviews at follow up will be blinded when assessing the primary and secondary outcome measures.

### Study settings and population

Rural Bangladesh is almost demographically and geographically homogeneous. Therefore, the selected sub-districts will be representative of two union parishads (5 villages) for the intervention group and two union parishads (5 villages) for the control group of the rural community of Bangladesh. Parents with children aged 1–4 years in these two groups will comprise the study population.

### Sample size calculation

The following formulae for hypothesis testing of the two group comparison was used [16] *N* = {Z1 – α/2 √2P̃ (1 – P̃) + Z1 – β √P1 (1 – P1) + P2 (1 – P2)}^2^ / (P1 – P2)^2^. P1 = Proportion of drowning in boy children aged under five years 55 % = 0.55; and P2 = Proportion of drowning in girl children aged under five years 45 % = 0.45 [17]. P̃ = (P1 + P2)/2 = 0.5 and adjusting for 10 % non-response *N* = 392+ 10 % of 392 = 431. A sample size of *N* = 431 in each study group would have 80 % power for a Chi-Square Test (*α* = 5 %, 2 *sided*) in order to detect this difference based on a calculation using G-Power. As union participants are nested within villages, a potential design effect needs to be considered. An assumed number of participants per village of 30 and an intra-cluster correlation coefficient of 0.05 could be expected. This would result in a design effect = 1+ (m-1) × ICC = 1+ (20–1) × 0.05 = 1.95. Multiplying this design effect by the “required size for an unnested sample (*N* = 431)” results in a required sample size of *N* = 840 per study group and a total of *N* = 1680 study participants.

### Intervention

The *MOBILE COACH* intervention aims to increase knowledge, safety awareness, safety behaviour and practices, and reduce the incidence of childhood drowning in Bangladesh. The SMS messages, images, videos and audio messages to reduce the incidence of drowning will be short, and in useful informal language, and, where possible, will be sent weekly (such as Friday, which is a holiday). These factors have been shown to have an impact and positively affect message acceptability when using SMSs for sexual health promotion [18]. The SMS, images, videos and audio messages will be developed by the researcher and the staff of the Centre for Injury Prevention and Research, Bangladesh. Mobile phones and the Internet are popular everywhere in Bangladesh and are well-liked by people, so we aim to determine whether they would be effective in reducing the number of children drowning.

### Technological background

The intervention programme named *MOBILE COACH* is fully based on the use of mobile technology. The programme used in the present study is similar to an earlier text-messaging programme for smoking cessation, which was successfully tested in two pilot studies [19]. All outgoing SMSs, videos, audio messages and images will be automatically recorded. All SMSs, images, videos and audio messages will be designed prior to the commencement of the broadcasting period.

### Intervention elements

The intervention programme consists of (1) assessment of the individual knowledge and awareness and practice about childhood drowning, and drowning related attitudes; (2) a weekly SMS and images for the assessment of knowledge, and awareness and practice about childhood drowning; (3) a monthly audio and video assessment of knowledge, and awareness and practice about childhood drowning; and (4) assessment to reduce the incidence of childhood drowning and risk factors for childhood drowning.

### Weekly SMS and images assessment

During the six-month intervention period, participants of the intervention group will receive one SMS and image per week for the assessment of knowledge and awareness and practice about childhood drowning. The weekly SMS and image assessment will be sent at a fixed point in time each week, 9:00 a.m. to 6:00 p.m., because 60 % of drowning events in Bangladesh happen during that time. All intervention participants will receive brief training concerning the use of the SMS and image intervention from the study assistants. On Friday morning of each week, the site study assistants will send an SMS and image to parents in the intervention group to enquire about their status and remind them of the availability of phone based support. The SMS will typically be 150–200 characters long and in Bangla language.

### Monthly audio and video assessment

Participants of the intervention group will receive one audio message and video per month for the assessment of knowledge and awareness, and practice about childhood drowning during the six-month intervention period. All the intervention participants will receive a brief training concerning the use of the videos and audio intervention from the study assistants.

### Control group

The study participants of the assessment only control group will not receive any of the intervention elements of the *MOBILE COACH* described above.

### Data collection method

#### Baseline survey

Fifteen study assistants and two supervisors will collect data from the two selected union parishad (15 villages) intervention groups and two union parishad (15 villages) control groups. The study assistants will home visit and collect data from the parents through face-to-face interviews using a set of pre-tested questionnaires. The baseline survey will be conducted one month before implementation of the respective intervention packages.

#### Injury surveillance

The study assistants for the surveillance areas will collect data by regular household visits at one-month intervals. The surveillance areas will be divided into 6 blocks, each consisting of around 100 to 150 households. For two blocks, one injury surveillance study assistant will be exclusively responsible for collecting data. In the first round of surveillance, the study assistant will be provided with a unique number for each of the households in a systematic manner, and will collect mobile phone numbers from each household. The study assistants will visit each household regularly at one-month intervals using surveillance instruments. Accordingly, they will visit approximately 20 households a day. The study assistants will conduct interviews with the parents of the family. The first time they visit the family, the study assistants will complete the Household Information Form. In each visit, the study assistants will check the injury surveillance system screening form. If any child is born or dies, the respective form will be completed. For each group area, one supervisor, the respective project co-coordinator, will be responsible to maintain the quality of the data.

#### End line survey

The end line survey for the intervention areas will be conducted in the last surveillance round. About 1680 households will be covered by 15 study assistants. Two supervisors will supervise the activities of the study assistants. The end line survey will use the same instruments as those used in the baseline survey and the same procedure will be followed to collect data. The survey will be conducted over 15 days.

#### Study duration

The duration of the study is six months. The activities of the intervention programme will commence in March 2015 and end in August 2015.

### Data analyses

The data will be entered into SPSS statistical package version 22.0.

The proportion, mean and standard deviation will be used to quantify the level of knowledge, awareness and practice before and after intervention.

The paired *t*-test will be used to examine the effectiveness of the intervention programme by comparing the pre-test and post-test score in respect of knowledge, awareness and practice.

The chi-square test will be worked out to determine the association of the socio-demographic, environmental and behavioural factors with childhood drowning.

In addition, the risk factors for childhood drowning with socio-demographic, environmental and behavioural factors will be determined using multivariate logistic analysis.

### Project monitoring and quality control

The Ministry of Science and Technology and Centre for Injury Prevention and Research, Bangladesh, collectively agrees with the planning, monitoring and evaluation processes employed in the project. The Ministry of Science and Technology, Bangladesh, has approved significant financial assistance.

### Outcomes

The outcomes of the study will be to determine the knowledge, awareness and behaviour practices about childhood drowning, as well as the risk factors, and reduce the incidence of childhood drowning. To determine the knowledge pertaining to childhood drowning, five questions about the knowledge of childhood drowning are provided. The questions will be answered YES or NO. A correct answer will score one mark and a zero mark will be given for an incorrect answer. The participants will be classified as having knowledge of drowning if they correctly answer all five questions. Based on those five questions, the total summated score will be calculated. Five questions will be used to measure the awareness about childhood drowning. Based on the five questions, the total summated score will be calculated. The behaviour practices will be divided into supervision practices and swimming ability. In addition, five questions will be used to measure the behaviour practices. Based on the five questions, the total score will be calculated. The study assistants will conduct the six-month follow up assessment by field interviews. The primary outcome will be the monthly assessment of increased knowledge, and safety awareness and practice about childhood drowning. The secondary outcome will be the assessment of reducing the incidence of children drowning to compare the baseline data and end line data, and also to determine the risk factors for childhood drowning. Finally, the outcome variables will be the effects of the mobile coach Intervention on the community in Bangladesh.

## Discussion

This study protocol is to determine the design of a cluster randomized controlled trial testing concerning the efficacy of mobile coach intervention to increase the knowledge and safety awareness, and practice about childhood drowning among the parents with children, and reduce the incidence of childhood injuries. This is the first controlled trial testing of the efficacy of mobile coach based childhood drowning intervention in a population consisting mainly of parents with children. Due to the realization of participant recruitment, it is also the first trial to test the efficacy of mobile coach based intervention in parents with children in terms of increasing their understanding pertaining to the risks of childhood drowning. Among the limitations of the study is that the majority of drowning cases go unrecorded, due to a lack of adequate survey data in hospitals and clinics, information bias and dependence on other data sources, such as police data and post-mortem data. Given that the mobile coach intervention is effective, it would provide an attractive, cost-effective model to increase the knowledge and awareness about childhood drowning and reduce childhood drowning in rural Bangladesh. The programme could be easily disseminated nationwide or via national prevention campaigns or internationally to other middle/low-income countries.
