# Erratum: Randomized controlled trial on drowning prevention for parents with children aged below five years in Bangladesh: a study protocol

**DOI:** 10.1186/s12889-015-2169-4

**Published:** 2015-09-15

**Authors:** Mosharaf Hossain, Kulanthayan K. C. Mani, Sherina Mohd Sidik, K. S. Hayati, AKM Fazlur Rahman

**Affiliations:** Department of Community Health, Faculty of Medicine and Health Science, University Putra Malaysia, 43400 UPM Serdang, Selangor, Malaysia; Department of Psychiatry, Faculty of Medicine and Health Science, University Putra Malaysia, 43400 UPM Serdang, Selangor, Malaysia; Department of Epidemiology, Bangladesh University of Health Sciences, Dhaka, Bangladesh

After publication of our article [1], it was brought to our attention that we had omitted to acknowledge that parts of the text were directly reproduced from the “Global report on drowning: preventing a leading killer” by the World Health Organization [2] cited as reference 14 in the article and from “Efficacy of a text messaging (SMS) based smoking cessation intervention for adolescents and young adults: Study protocol of a cluster randomised controlled trial” by Haug et al. [3]. We apologise for any inconvenience caused.
